# The Poor-Law Midwifery Schools

**Published:** 1908-02-01

**Authors:** 


### Nursing Administration.

### THE POOR-LAW MIDWIFERY SCHOOLS.

It has been seen that a good deal of indignation has been wasted in attributing the shortage of pupils sent up for examination in midwifery by Poor-law infirmaries and workhouses to the action of the Central Midwives Board in refusing to recognise as training schools for midwives institutions which can only offer material for training one or two midwives in the year. The Central Midwives Board has effectually vindicated itself from the charge by supplying a list of the institutions refused the hall mark of recognition during the year 1907. These institutions are ten in number, and the number of deliveries average 26.8 per annum, the highest being 63 and the lowest 8. Since the rules prescribe that "each candidate for examination must have attended and watched the progress of not fewer than twenty labours, making abdominal and vaginal examinations during the course of labour, and personally delivering the patient," it is clear that the extreme number of candidates who could have been prepared in all these institutions was thirteen; but as in four instances the number of deliveries fell below twenty, this number would undoubtedly have been still further reduced in the actual working. This vindication of the Central Midwives Board does not, however, make the situation less grave. It rather adds to the problem of increasing the supply of midwives. So long as the easy belief was entertained that an unlimited supply of midwives could be turned out from Poor-law establishments so soon as red tape would permit, the outlook was tolerably hopeful, because even red tape has its limits, and where everyone desires a rapprochement it cannot long delay to take place. But this view of the matter has now been swept away. It is neither probable nor desirable that large midwifery schools will ever come to be associated with Poor-law institutions save in a few exceptional instances. But the grave part of the matter is that no extension of voluntary midwifery schools can be looked for either. It is now many years since any new lying-in hospital of importance has been added to our voluntary charities. The ease with which women are able to receive attention in their own homes, the success of district midwifery in homes of squalor and destitution renders such additional hospitals for lying-in mothers superfluous. The present voluntary midwifery schools are doing as much as they possibly can, and it is vain to expect from this quarter there can be any increase in the number of candidates for examination. How, then, can the urgent need for more pupil midwives be met? It is in this direction that we look for guidance to the Central Midwives Board. And it must be added that we look in vain. The Midwives Act of 1903, under which the Central Midwives Board came into being, had a twofold object. It aimed at the repression of incompetent midwives, but no less clearly it aimed at replacing them by trained women. Whatever tends to encourage the systematic training of women for this branch of the public service lies within the province of the Central Midwives Board, and the public has a right to expect a forward policy from this important new body. Unhappily, the Board has from the first been dominated by a cautious and reserved spirit which may almost be called suspicious, and it appears to be so completely absorbed in the duty of keeping at bay unworthy candidates that it omits altogether the no less obvious duty of smoothing the way for the trained candidates so urgently required. In no particular is this more marked than in the matter of conferring the mark of "recognition" on institutions which claim to train midwives for examination. The vexatious course has been adopted by the Board of laying down no special regulations which must be complied with by institutions aiming at "recognition." Each one is compelled to apply on its own merits, nor, if rejected, is information always accorded as to the reasons which have guided the Board in coming to an adverse decision. Does the Board realise that it is irritating to the last degree for an influential establishment to be compelled to make application in the dark and be met with blank refusal? Multiply such refusals by forty or fifty in the course of the last three years, and the reason for the growing sense of indignation on the part of Poor-law institutions is fully accounted for. The mode of procedure is neither courteous nor reasonable. For clearly the Board have their own regulations for "recognition." The matter is one of too great importance to be left to the varying views of the members of the Board who may chance to be present at any given meeting. One of these regulations has been brought to light by the Bethnal Green Guardians, who state in their last Triennial Report they were officially informed that their application for the recognition of the Bethnal Green Infirmary had been rejected on the ground that the Central Midwives Board "had made a rule not to approve for that purpose any institution in which the total number of deliveries per annum was less than 75." Why has this rule not been plainly set forth from the very beginning? Why are not the other vital conditions attached to the recognition of training schools for midwives made public? If it be deemed possible that all rules might be complied with while still the general conditions were unsuitable for good training, it would be easy to publish clear particulars of the rules enforced and to state that the Midwives Board can only recognise as training schools institutions which comply with these regulations and are approved by the Board after inspection by their own inspector. Had this straightforward course been adopted from the first, all the deplorable friction which has arisen between the Poor-law institutions and the Central Midwives Board would have been avoided. There is nothing more disastrous to adopt for a public body than a secretive policy.

(*To be continued.*)

